# Using an expert judgment response matrix to assess the risk of groundwater discharges from remediated fuel spill sites to the marine environment at sub‐Antarctic Macquarie Island, Australia

**DOI:** 10.1002/ieam.4382

**Published:** 2021-02-10

**Authors:** Catherine K King, Jane Wasley, Jessica Holan, Jeremy Richardson, Tim Spedding

**Affiliations:** ^1^ Australian Antarctic Division Kingston Tasmania Australia

**Keywords:** Direct toxicity assessment, Risk assessment, Petroleum hydrocarbons, Degraded fuel, Marine invertebrate toxicity

## Abstract

This study assesses toxicity of groundwater from remediated fuel spill sites, as the final phase of an environmental risk assessment of contaminated sites at sub‐Antarctic Macquarie Island, Tasmania, Australia. To complement previous terrestrial ecotoxicological research, we determine risk to marine environments from residual biodegraded hydrocarbon contaminants in groundwater discharges. Direct toxicity assessments were conducted on 7 composite groundwater test solutions, adjusted to ambient seawater salinity. Eleven native marine invertebrates (from varied taxa: gastropods, bivalves, flatworms, amphipods, copepods, isopods) were exposed and observed for up to 21 d. Lethal time estimates (LT10, LT50) showed sensitivity was time dependent (LT10s = 4–15 d) and variable between species. Three species showed no response to any test solution, and most species did not respond for up to 5 d. Data were interpreted using an expert judgment response matrix with multiple lines of evidence to predict risk. No consistent patterns in the relative toxicity of test solutions, based on polar or nonpolar hydrocarbon concentrations, were identified. Although toxicity was observed in some species, this was only under worst‐case conditions of undiluted, continuous, extended exposure. Natural dynamics of the site, including low groundwater discharge rates, high rainfall, and a highly energetic receiving environment, ensure groundwater is rapidly diluted and dispersed. In this context, and based on site conditions at the time of testing, these toxicity assessments provide robust evidence that residual contamination in groundwater at remediated sites at Macquarie Island is unlikely to represent a risk to the adjacent marine communities tested. *Integr Environ Assess Manag* 2021;17:785–801. © 2020 The Authors. *Integrated Environmental Assessment and Management* published by Wiley Periodicals LLC on behalf of Society of Environmental Toxicology & Chemistry (SETAC)

## KEY POINTS

- A novel and transparent expert judgment response matrix was used to assess and interpret responses of native marine invertebrates in direct toxicity assessments to groundwater discharge from remediated fuel spill sites at sub‐Antarctic Macquarie Island, Tasmania, Australia.
- Response to exposure was slow, with a minimum of 4 d required to elicit a response in any test species.
- Toxicity of test solutions to biota was not able to be attributed to discharge chemistry, including concentration of both polar and nonpolar hydrocarbons.
- Residual contamination in groundwater was deemed unlikely to cause risk to coastal marine communities, given the overall low toxicity of test solutions, the slow responses of test species, and the highly energetic receiving environment.

## INTRODUCTION

Macquarie Island (Tasmania, Australia) is a remote sub‐Antarctic island in the Southern Ocean that lies approximately halfway between Australia and the Antarctic continent. It is a World Heritage‐listed reserve and supports large populations of wildlife such as seals, penguins, and seabirds. A research station has been located at the northern end of the 34‐km‐long island since 1948 (at 54°29′58.2″S, 158°56′09.2″E). During these 7 decades of occupation, the footprint of the station has been subject to environmental incidents, including fuel spills associated with bulk fuel storage and use. An oil spill in the marine environment also occurred as a result of the grounding of the ice breaker, *Nella Dan*, during resupply operations in 1987. Since 2003, the Australian Antarctic Division has overseen a program of applied remediation and research focusing on 2 of the main fuel‐contaminated sites within the station footprint, the Fuel Farm and the Power House. Terrestrial environments at these sites have a complex history of contamination associated with station activities. Elevated hydrocarbon contamination was documented at these sites more than 25 y ago (Deprez et al. 1994), and since that time at least 1 other fuel spill event (in 2002) has been reported (Rayner et al. 2007). Active remediation was carried out on site from 2009 to 2016. The main in situ bioremediation strategy implemented was installation of air and nutrient injection arrays, which provided elevated O_2_ and nutrient levels to soils to enhance microbial degradation of hydrocarbon contaminants. Nutrient additions were applied during discrete events across a 5‐y duration and included delivery of liquid solutions via the injection arrays (urea, ammonium nitrate, calcium nitrate, and ammonium polyphosphate) and solid slow‐release fertilizer (Agroblen) at the soil surface. During remediation, soil and shallow groundwater samples were collected from within the remediation zones to monitor surface and subsurface contaminant concentrations and dynamics as well as a range of physicochemical traits. A permeable reactive barrier was installed in 2014 to capture and treat hydrocarbon‐contaminated groundwater (Freidman et al. 2017). Groundwater monitoring included regular sampling from piezometers installed in soils (<2 m depth), as well as at water seeps discharging from exposed escarpment soils along the coastal foreshore. Further postremediation groundwater assessment was facilitated in foreshore areas via the repeated sampling of additional shallow piezometers installed directly into exposed foreshore bedrock.

In conjunction with the bioremediation and monitoring programs, a detailed risk evaluation was conducted to determine ongoing risk to the environment at the sites. Site‐specific soil ecotoxicology studies were used to predict the ecological risks to soil communities posed by exposure to hydrocarbons, with the ultimate goal to derive site remediation targets for soils. To date, the focus of this ecotoxicology research has been on assessing the impacts of fuel contamination in the terrestrial ecosystem (Errington, King, Wilkins et al. 2018). From this work, we have obtained a robust understanding of the impacts of hydrocarbons on a range of soil biota, including plants (Bramley‐Alves et al. 2014; Macoustra et al. 2015), invertebrates (Mooney et al. 2013; Wasley et al. 2016; Errington, King, Houlahan et al. 2018; Mooney et al. 2019), and soil microbes (Schafer et al. 2007; van Dorst et al. 2014, 2020).

The risk of hydrocarbon contamination to terrestrial biota at the site is therefore relatively well understood; however, there has been no assessment of potential risks to adjacent marine communities. The only studies that have examined the effect of hydrocarbons on marine communities at Macquarie Island were done in association with the 1987 grounding of the *Nella Dan* (Smith and Simpson 1995, 1998). Although the marine environment is less directly exposed to terrestrial hydrocarbon sources, on‐site groundwater monitoring has identified seepages of fuel‐contaminated water discharging via coastal cuts onto the shoreline margins of the contaminated sites. Fuel and other contaminants are therefore at risk of migrating from these groundwater seepages to the coastal marine environment. Whether this contamination source poses a risk to the Macquarie Island marine environment is unknown. Preliminary toxicity assessments using standard Microtox bioassays indicated measurable acute toxicity to the marine bacterium from groundwater. However, this standard bioassay is of limited ecological relevance for Macquarie Island and may not reflect the sensitivity and response of local biota, hence the need for further site‐specific risk analysis using local marine species.

Direct toxicity assessment (DTA) is commonly used to inform site‐specific risk assessments and is recommended in the current Australia–New Zealand water quality guidelines (ANZG 2018). This approach tests real‐world environmental samples to determine the combined effects of contaminants and the overall toxicity of complex mixtures, generally without explicitly attributing observed toxicity to specific chemicals and concentrations (Gruiz et al. 2016). Direct toxicity assessments are of the highest environmental relevance because they assess all interactions among contaminants, physicochemical properties, and water and soil phases, thereby combining the effect of all stressors present in a sample as a combined exposure. Direct toxicity assessments are regularly used in the assessment of industrial sites such as mines (e.g., Trenfield et al. 2019; Angel et al. 2020), groundwater at fuel release sites (e.g., Patterson et al. 2020), and contaminated groundwater discharge into waterways (e.g., Hunt et al. 2009) to determine the appropriate dilution of discharges and to derive site‐specific guidelines.

Direct toxicity assessments typically test dilutions of a sample to determine critical effect concentrations (using % as units). In the present study, however, we test each environmental sample at its maximum concentration only, and determine critical exposure duration (as lethal time, or time to death). Lethal time estimates are used where duration of exposure is an additional criterion of interest, and they have been used to assess the toxicity of a range of substances to marine biota (Jones et al. 2006; Finch and Stubblefield 2016). Lethal time estimates are also commonly used in research on invertebrate pest management where understanding the time for a treatment to be effective is as important as understanding the concentration that causes lethality (e.g., Hansen et al. 2004; Mansour and Gad 2010). Similarly, in the present study we consider the time taken for a contaminated groundwater to cause toxicity to be essential information in the risk assessment framework where the characteristics of the receiving environment influence exposure dynamics.

The objective of the present study was to conduct ecotoxicological tests on native marine biota to assess potential risk of exposure to contaminated groundwater that migrates from fuel‐contaminated sites on land into the marine receiving environment. The present study was conducted during the 2017–2018 summer field season at Macquarie Island, 1 y after remediation activities were ceased on site. Direct toxicity assessment methods were used to assess the toxicity of field collected groundwater to 11 local indigenous species of marine invertebrates and to a bioluminescent bacteria using the standard Microtox test. Toxicity test methodologies used in DTAs were based largely on protocols developed previously for Macquarie Island marine biota by Holan et al. (2016, 2017, 2018, 2019) and Lewis et al. (2016), whose work constitutes a significant contribution to our understanding of marine ecotoxicology in sub‐Antarctic environments. These protocols were modified in the present study to determine time to death as opposed to lethal concentrations as a measure of relative toxicity.

We present and interpret toxicity data using an expert judgment response matrix that incorporates multiple lines of evidence to predict the risk to the receiving invertebrate marine communities at Macquarie Island from the migration of residual contaminants from terrestrial sites. Results from the present study contribute scientifically robust lines of evidence that will inform an overall risk assessment of fuel‐contaminated sites at Macquarie Island. Other lines of evidence in the overall risk assessment (not reported here) include ecotoxicological responses of terrestrial biota, ecological surveys, and chemical risk modeling. Together, these assessments will be used to determine whether the site poses any ongoing environmental risk.

## METHODS

### Study sites

**Figure 1:**
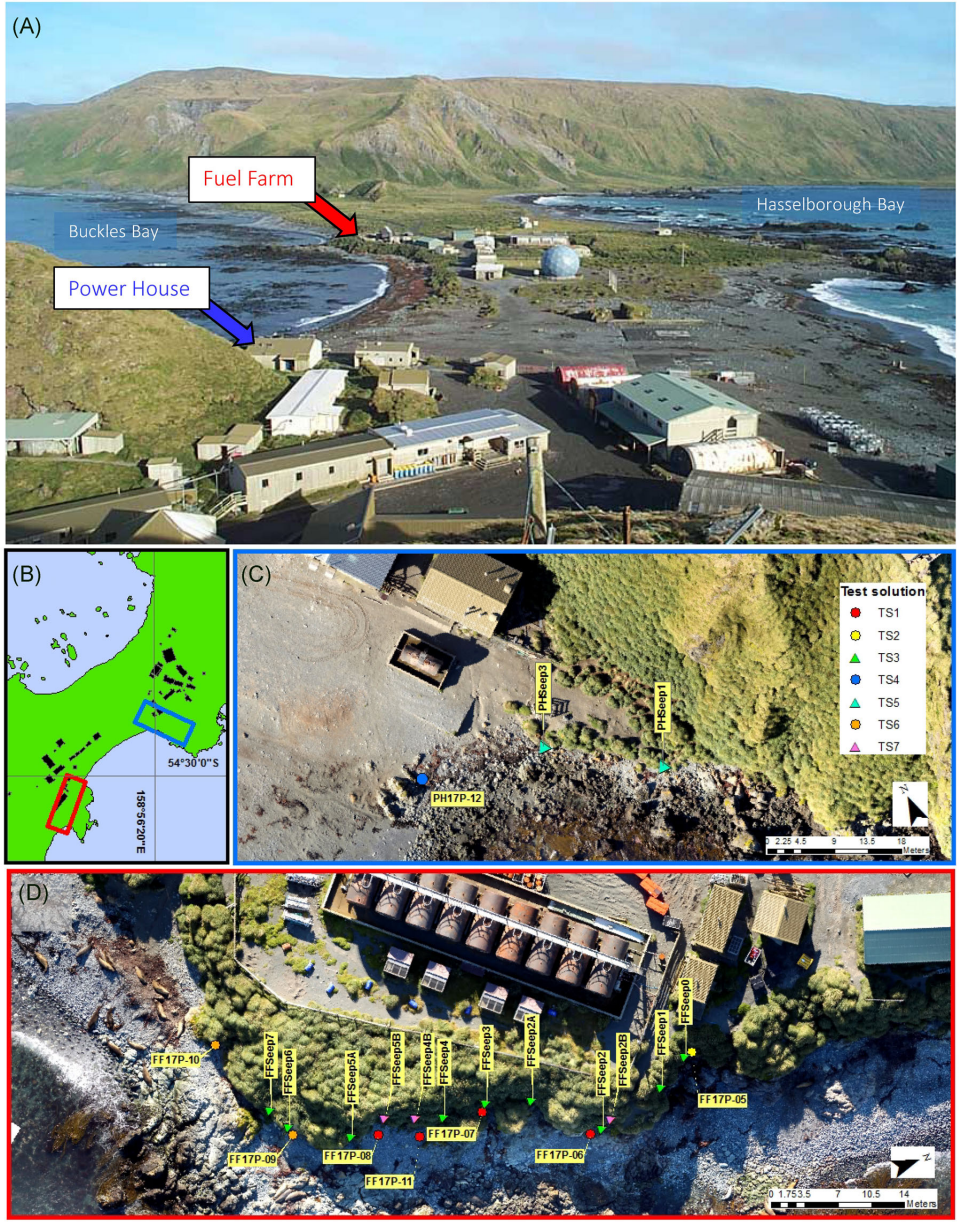
Macquarie Island study sites and sampling locations: station area and environment (**A**); map of station area and infrastructure (black) showing relative location of sites Fuel Farm (red) and Power House (blue) (**B**); Fuel Farm (**C**) and Power House (**D**) showing monitoring points used to collect groundwater samples for test solution (TS) preparation: piezometer (circle) and seep (triangle). Refer to King et al. (2020) for further detail on labeled monitoring points.

Two areas within the vicinity of the Macquarie Island research station, the Fuel Farm and the Power House, were the focus of the present study. These sites are located close to the coastline at Buckles Bay, as shown in Figure 1 (for a detailed map of the station area, see King et al. 2020).

### Foreshore groundwater sampling

Groundwater monitoring (chemical analysis) from previous seasons (data not shown) was used to identify 22 monitoring points across the 2 sites to be sampled to produce a series of composite groundwater solutions for testing (Figure 1). This subset of monitoring points was selected to target discharges in the vicinity of the foreshore of the 2 sites that reported contamination in previous monitoring events. The composite solutions were designed to represent the range of complex contaminant mixtures that would be released from across the sites to the adjacent marine environment. Samples were grouped based on site, similarities in the type of groundwater discharge (i.e., seep or fractured bedrock aquifer), and on their chemical properties in terms of composition and concentrations (Figure 1, Supporting Information Table S1). Because the coastal margin was more expansive at the Fuel Farm than at the Power House, the majority of monitoring points were located at the Fuel Farm site.

Groundwater was collected via piezometers or from coastal seeps. For piezometers positioned within the bedrock, as was the case at the Fuel Farm site, groundwater was sampled from deep in the aquifer, providing samples with long residence times. In contrast, groundwater from seeps originates from the soil layer at the bedrock–soil interface and has far shorter residence times. Samples were collected from 7 foreshore piezometers and 12 seeps at the Fuel Farm, and from 1 piezometer and 2 seeps at the Power House (see Supporting Information Table S1 for details).

Piezometer and seep collection points were purged dry prior to water samples being collected. Water from piezometers was collected via ½‐inch‐diameter low‐density polyethylene (LDPE) tubing connected to a peristaltic pump (Solinst model 410) by silicon tubing. The peristaltic pump silicon tubing was decontaminated between locations in a Decon‐90 surfactant solution prior to double rinses in deionized water (Milli‐Q, 18 MΩ.cm‐1; Merck Millipore), and new LDPE tubing was used for each piezometer. Water from surface seeps was directly sampled via new 50‐mL plastic syringes fitted with a 0.45‐µm filter. Personnel wore new nitrile gloves for each sampling point to prevent cross‐contamination between samples. Samples were collected directly into new 500‐mL amber glass bottles and returned to the laboratory for test solution preparation.

A maximum of 5 L was collected from each of the monitoring points, depending on the volumes required for preparation of composite test solutions. The time taken to obtain each groundwater sample was variable, from 1 to 12 h, depending on the recharge rate of the individual seep or piezometer, and on the volume required for the composite sample.

Physicochemical properties of water samples were measured on site using a multiparameter water monitoring meter (Aquaread Aquameter with Aquaprobe AP‐2000) to determine dissolved oxygen (DO), total dissolved solids (TDSs), salinity, temperature, electrical conductivity (EC), oxidation–reduction potential (ORP), pH, and turbidity. The Aquaprobe was calibrated each morning before use as per the manufacturer's instructions. Samples were then immediately returned to the laboratory and stored frozen (–18 °C) until composite test solutions were prepared, immediately prior to toxicity testing.

Groundwater sampling was repeated in preparation for 2 rounds of subsequent toxicity tests. Monitoring points were sampled during low tide on 23 to 29 November 2017 (Round 1) and 18 to 20 December 2017 (Round 2). Each round provided a “snapshot” of contamination and site conditions at the time of sampling.

### Test solution preparation

All glassware and equipment used for the preparation and storage of test solutions was either new or was washed by soaking in Decon 90 for ≥24 h, followed by extensive rinsing with Milli‐Q.

Test solutions (TSs) were prepared by selectively compositing the 22 piezometer and seep groundwater samples into 7 salinity‐adjusted composite test solutions (TS1–TS7) for use in toxicity tests. The 7 test solutions were each prepared to a total volume of 5 L and adjusted to the local ambient salinity of marine waters. A single groundwater sample was used for TS2 and TS4; all other TSs (1, 3, 5, 6, and 7) were composites, comprised of equal volumes of multiple groundwater samples (Supporting Information Table S1).

Seawater (SW) was used both as a control water in tests and to prepare hypersaline brine (HSB) for test solution salinity adjustment. Seawater was collected locally from Landing Beach or Hasselborough Bay, both of which are located away from obvious signs of contamination and from the influence of the station. Seawater was collected in clean high‐density polyethylene containers, returned to the station laboratory, and stored at 4 °C in the dark until used in tests or to prepare HSB. The HSB was prepared by freezing containers of SW, allowing them to partially defrost, then collecting the concentrated brine. Salinity of each composite sample was adjusted to the test salinity of 33‰ to 34‰ by the addition of appropriate volumes of HSB. This salinity adjustment resulted in dilution of composite samples (to concentrations of 76%–88%), depending on the salinity of the composite sample, salinity of the HSB, and the volume of HSB therefore required to achieve the test salinity. An HSB control was also prepared by the addition of Milli‐Q water to achieve the test salinity.

Prepared test solutions were gently decanted into clean 500‐mL amber glass bottles with foil septa. Two bottles, each containing 250 mL of test solution, were allocated as subsamples for additional analysis and testing upon return to Australia. One subsample bottle was acidified via the addition of 0.5 mL of a 5‐M sulfuric acid solution and was used for total recoverable hydrocarbon (TRH) analysis. The second subsample bottle was used for Microtox assays and nutrient analysis. The bottles were frozen (–18 °C) for the duration of their extended storage and transport (approximately 5 mo) and analyzed by the receiving laboratory within 48 h of thawing. All remaining bottles were stored frozen (–18 °C) to minimize contaminant degradation until used in invertebrate toxicity tests on site.

### Physicochemical analysis of test solutions

Immediately prior to use in toxicity tests, test solutions were thawed and physicochemical properties measured using the multiparameter water monitoring meter to determine DO, TDS, salinity, temperature, EC, ORP, pH, and turbidity (as per the water quality assessments during groundwater collection on site). Salinity was also measured using a salinity meter (WTW multi 3410).

The subsamples of each test solution were returned to Australia for analysis of nutrient (N) and TRH concentrations with and without silica gel cleanup, to enable both polar and nonpolar hydrocarbon concentrations to be determined. Standard TRH methods, without silica gel cleanup, quantify total hydrocarbons, without discrimination between polar and nonpolar hydrocarbons. Silica gel cleanup removes polar hydrocarbons and measures concentrations of nonpolar hydrocarbons only, allowing any difference in the 2 measures to be attributed to polar hydrocarbons. This procedure allows interrogation of the TRH signal, to determine concentration of hydrocarbons that are petrogenic (nonpolar) versus degradation products (metabolites from petrogenic sources and/or sources of natural organic matter). Silica gel cleanup is therefore important for the interpretation of TRH chemistry within samples (Zemo et al. 2017; Patterson et al. 2020). Analyses were conducted by Australian Laboratory Services Environmental Division, Melbourne (Australia; NATA Accredited Laboratory Nr. 825, site nr. 13778). Standard methods were compliant with the National Environmental Protection Measure Schedule B(3) (NEPC 2013; NEPM Method 506.1), and were based on American Public Health Association (APHA 2017; Method 4500) and United States Environmental Protection Agency (USEPA 2015; SW‐846 Test Method) protocols. Quality assurance/quality control (QA/QC) details and the laboratory results report are provided (King et al. 2020).

Nitrogen was analyzed in nonacidified subsamples using direct colorimetry by discrete analyzer. The standard methods referenced for analysis were APHA 4500‐NH3 G for ammonia as N, APHA 4500‐NO2‐B for nitrite as N, and APHA 4500‐NO3‐F for nitrite and nitrate as N, with the latter conducted for combined oxidized N (NO_2_+NO_3_) after chemical reduction of nitrate to nitrite prior to quantification by discrete analyzer. Nitrate as N was calculated as the difference, following APHA 4500‐NO3‐F.

The TRH was analyzed in acidified subsamples. Subsamples were prepared by separating funnel extraction of liquids following USEPA SW‐846 Test Method 3510B, with 100 to 1000 mL of sample transferred to a separating funnel and serially extracted 3 times using 60 mL dichloromethane per extract. The resultant extracts were combined, dehydrated, and concentrated for analysis. Any sediment remaining in containers was excluded.

Analysis of semivolatile TRH in sample extracts was done using capillary gas chromatography/flame ionization detector (GC/FID) to determine the >C10–C40 fraction; using an established 5‐point calibration curve of *n*‐alkane standards) and with silica gel cleanup to remove polar compounds (including nonpetroleum hydrocarbon interferences) to provide a total estimate following USEPA SW‐846 Test Method 8015C; using alkane standards over the range >C10–C40 following NEPM Method 506.1.

Aqueous samples for analysis of volatile organic compounds, including benzene, toluene, ethyl benzene, and xylenes collectively known as (BTEX), were prepared in accordance with USEPA SW‐846 Test method 5030B. Volatile compounds are transferred to a sorbent column by sparging a 5‐mL portion of sample (or diluted sample) with an inert gas, which is purged onto the sorbent column. The sorbent column is then heated and back flushed with an inert gas to desorb the compounds for determination via capillary GC–MS in accordance with USEPA SW‐846 Test method 8260B, with quantification using an established 5‐point calibration curve.

### Microtox toxicity tests

Microtox assays were conducted by Australian Laboratory Services, Scoresby (Australia; NATA Accredited Laboratory Nr. 992, site nr. 989), using the remainder of the nonacidified subsample. This standard in vitro testing system measures luminescence response of the marine bacterium *Allivibrio fisheri* following exposure to contaminants. The EC50 values were based on percent dilution of the test solution and were determined after a 15‐min exposure with the following interpretation provided by the laboratory, as test solution dilution: ≤25% extremely toxic, 25% to 50% highly toxic, 51% to 75% moderately toxic, 76% to 100% slightly toxic, >100% no measurable toxicity.

### Invertebrate toxicity tests

#### Collection of test species

A total of 11 marine invertebrate species from a range of taxonomic groups was used across the 2 rounds of testing. Species tested were amphipods *Paramoera* sp. and *Parawaldeckia kidderi*, isopod *Exosphaeroma gigas*, copepods *Zaus* sp. and *Tigriopus angulatus*, gastropods *Laevilitorina caliginosa* and *Macquariella hamiltoni*, bivalves *Gaimardia trapesina* and *Lasaea hinemoa*, and flatworms *Obrimoposthia ohlini* and *Obrimoposthia wandeli*. All test species are common to marine environments at Macquarie Island and were collected at low tide from either Garden Bay on the east coast or Hasselborough Bay on the west coast (see King et al. 2020 for station area map). For all test species, smaller individuals within the population were targeted in collections, and the size of individuals in the tested cohort relative to the total population was noted (Table 1). Invertebrates were kept in temperature‐controlled cabinets at 6 °C (±1 °C) and used in toxicity tests within 2 d of collection.

**Table 1:** Marine invertebrate species tested in this study to assess the toxicity of groundwater discharges from remediated fuel spill sites at sub‐Antarctic Macquarie Island, Tasmania, Australia, including details of collection, size range, and test conditions

Species	Taxonomic group	Collection location	Collection habitat	Body length (mm), relative size^a^	Test conditions		Observation days
					Test round	Test volume (mL)	
*Paramoera* sp.	Amphipod	Garden Bay, East Coast	High intertidal splash pools	8.2 (1.3), medium	1	150	1, 2, 4, 7, 10, 14
*Parawaldeckia kidderi*	Amphipod	Garden Bay, East Coast	High intertidal splash pools	5.2 (0.7), small	2	120	4, 7, 10, 14, 21
*Exosphaeroma gigas*	Isopod	Garden Bay, East Coast	High intertidal under rocks	2.9 (0.3), small	1	100	1, 2, 4, 7, 10, 14
*Zaus* sp.^b^	Copepod	Hasselborough Bay, West Coast	Rocky outcrop	0.8 (0.2), medium	1	30	1, 2, 4, 7, 10, 14
					2	30	1, 2, 4, 7, 10, 14
*Tigriopus angulatus* c	Copepod	Garden Bay, East Coast	High intertidal splash pool	1.4 (0.2), medium	1	50	1, 2, 4, 7, 10, 14, 21
					2	50	4, 7, 10, 14, 21
*Laevilitorina caliginosa*	Gastropod	Garden Bay, East Coast	High intertidal under rocks	3.2 (0.7), medium to large	1	100	1, 2, 4, 7, 10, 14
					2	120	4, 7, 10, 14, 21
*Macquariella hamiltoni*	Gastropod	Hasselborough Bay, West Coast	Rocky outcrop	2.6 (0.5), medium to large	1	90	1, 2, 4, 7, 10, 14
					2	120	1, 2, 4, 7, 10, 14
*Gaimardia trapesina*	Bivalve	Hasselborough Bay, West Coast	Rocky outcrop	2.5 (0.5), small	1	150	1, 2, 4, 7, 10, 14, 21
					2	150	4, 7, 10, 14, 21
*Lasaea hinemoa*	Bivalve	Garden Bay, East Coast	High intertidal in crevices to shore	2.3 (0.3), small	2	90	4, 7, 10, 14, 21
*Obrimoposthia ohlini*	Flatworm	Garden Bay, East Coast	High intertidal splash pools	5.4 (1.2), medium to large	2	90	4, 7, 10, 14, 21
*Obrimoposthia wandeli*	Flatworm	Garden Bay, East Coast	High intertidal under rocks	3.4 (0.7), medium	1	50	1, 2, 4, 7, 10, 14
					2	90	4, 7, 10, 14, 21

^a^ Mean (SD); observation of relative size of tested cohort within the population.

^b^ Includes gravid and nongravid individuals.

^c^ Nongravid individuals only.

#### Toxicity tests

A site‐specific DTA approach was used to assess the effects of test solutions on test species. The DTA aimed to investigate the immediate impact of “undiluted” groundwater discharges (maximum concentrations, as close to 100% as possible, depending on the volume of HSB used for salinity adjustments) on biota at the point of discharge, as a worst‐case scenario. Responses to test solutions were compared to responses in HSB and SW controls.

Two rounds of toxicity tests were conducted to enable some examination of temporal variation in the toxicity of test solutions and the response of biota. Round 1 tests started on 4 December 2017 (Round 1A) and 7 December 2017 (Round 1B). Round 2 tests started on 23 December 2017. Round 1 tests included all 7 test solutions; Round 2 tests included 6 test solutions, with TS5 omitted due to low seepage rates that prevented adequate volumes of groundwater being collected from monitoring points.

Test methodologies based on previous work were used for 7 species: *Zaus* sp., *T. angulatus*, *Laevilitorina* *caliginosa*, *M. hamiltoni*, *G. trapesina*, and *O. ohlini* (Holan et al. 2016, 2017, 2018, 2019), and *E. gigas* (Lewis et al. 2016). For the remaining 4 species, test methodologies were developed during the present study (full test method details are available in King et al. 2020). For all test solutions there were 5 replicate vials, and for both SW and HSB controls, there were 6 replicate vials. A total of 10 individuals was added to each replicate vial at the start of tests. Test volume was 30 to 150 mL depending on the species tested (Table 1). Tests were conducted at 6 °C (±1 °C) and were static, without water renewals, to represent continuous press exposure to the contamination signature of a test solution at a single point in time and at the highest concentration possible in a single flushing event. Exposure duration was 14 or 21 d, depending on the test species, with observations of survival made periodically through time (Table 1). Sublethal endpoints were also observed for some biota, but were more variable and less reliable, and are not presented here (data available in King et al. 2020). A subsample of individuals of each species (minimum of 10) was preserved in 70% ethanol and later examined under a microscope to measure total body length (Table 1).

#### Statistical analysis

Sensitivity to each test solution was quantified as critical exposure duration (time to death in *x*% of the tested population). Data were analyzed with the R statistical program, version 3.6.2 (R Core Team 2019) using the drc package (Ritz et al. 2015).

Critical exposure duration models (using traditional concentration–response models) were fitted to the effects data (% survival) for each test solution and control. A selection of models from the drc package was fitted to effects data: 3‐, 4‐, and 5‐parameter log‐logistic models (LL.3, LL.4, and LL.5), the 3‐parameter log‐logistic model (LL.3) with the upper asymptote set to 100, and two 4‐parameter Weibull functions (W1.4 and W2.4). Best model fit was assessed via visual inspection and by comparison of Akaike information criterion (AIC) values. In most cases, the modified 3‐parameter log‐logistic model, with upper asymptote set to 100, was selected as the best model fit (lowest AIC). Selected models were used for subsequent critical exposure duration derivation, in which lethal time (LT) to reach a 10% or 50% decline in survival (LT10, LT50) was calculated.

### Expert judgment response matrix

In most ecotoxicological studies, toxicity is determined using modeled response estimates. These are most reliable when estimates and their associated confidence intervals show a clearly defined response relative to control (ideally from 0% to 100%) over a concentration gradient or time series. In contrast, in the present study, multiple lines of evidence were used to provide a summary of the relative risk of each test solution and the relative sensitivity of each invertebrate test species. The use of multiple lines of evidence was required because many partial responses were observed, and modeled LT estimates alone were not consistently adequate for determining response.

Multiple lines of evidence are presented as criteria to provide a decision framework that allows for an objective and transparent assessment of response. Five response criteria were developed based on expert judgment (Table 2). Each criterion was assigned a weighting value between 1 and 4, also set by expert judgment, which reflects relative importance in assessing overall response. Each criterion was scored 1 if met (response), 0 if not met (no response), or 0.5 if borderline or unclear. A response score was calculated for each of the 110 species and test solution combinations using the sum of the 5 weighted criteria, expressed as a percentage, as the basis to determine whether response had been observed in each case. Response scores of 65% or greater were, by expert judgment, considered to provide suitable evidence of response.

**Table 2:** Description of the 5 response criteria selected using expert judgment, which were used to assess toxicity of each test solution to marine invertebrates^a^

Criterion	Description	Weighting value	Data source
1	By visual inspection of raw data plots showing survival through time (Supporting Information Figure S1), response in test solution through time is distinct from response in HSB and SW controls (response observed at a minimum of 1 time point).	4	Figure 2, Supporting Info. Figure S1
2	Calculated difference between mean survival in test solution and HSB control is ≥20% (i.e., HSB control response − test solution response >20%) at end of test. Assessed as borderline if 15%–20% difference.	3	Table 5, Supporting Info. Table S4
3	Mean survival in test solution is <80% at end of test. Borderline if 80% to 85%.	2	Table 5, Supporting Info. Table S4
4	Using lethal time to 10% response (LT10) modeled estimates, test solution LT10 ± 95% CI estimate range is below the HSB control LT10 estimate range (i.e., LT10 values do not overlap). Borderline if test solution 95% upper limit is within the HSB control estimate range, but test solution mean is below the HSB control 95% lower limit.	1	Table 4, Supporting Info. Table S3, Figure 3, and Supporting Info. Figure S2
5	Using lethal time to 50% response (LT50) modeled estimates, test solution LT50 ± 95% CI estimate range is below the HSB control LT50 estimate range (i.e., LT50 values do not overlap). Borderline if test solution 95% upper limit is within the HSB control estimate range, but test solution mean is below the HSB control 95% lower limit.	1	Table 4, Supporting Info. Table S3, Figure 3, and Supporting Info. Figure S2

HSB = hypersaline brine; SW = seawater.

^a^ Each criterion was assigned a weighting value of 1 to 4 by expert judgment, with values reflective of the relative importance in assessing response. Data source referred to for evaluation of each criterion is provided.

## RESULTS AND DISCUSSION

### Physicochemical properties of test solutions

Final concentrations of test solutions after salinity adjustment to 33‰ to 34‰ ranged from 76% to 88% of the original composite groundwater solutions (Table 3). Full characterization of physicochemical parameters is shown in Supporting Information Table S2, and these were mostly within expected ranges for Southern Ocean SW. The exception was pH, which was low in some test solutions (6.14–7.38), especially in Round 2, in which the SW used as the control and to prepare HSB for test solution salinity adjustment had a pH of 7.5 (Supporting Information Table S2). As a consequence, the pH of all test solutions in Round 2 was relatively low. For most chemical analytes measured, concentrations were below laboratory limit of reporting (Supporting Information Table S2), with the exception of petroleum hydrocarbons and nutrients, which are shown in Table 3. Concentrations of these chemicals were relatively consistent between rounds for all test solutions (Table 3, Supporting Information Table S2). Hydrocarbon concentrations in the >C10 to C40 range varied between test solutions, with most below 500 µg/L. The highest concentration reported was 810 µg/L in TS7 (Table 3). Most of the measurable hydrocarbons in the >C10 to C40 range were within the F3 fraction (>C16–C34; Supporting Information Table S2). After silica gel cleanup, hydrocarbons in all test solutions were below detection (Table 3). This confirmed that all reported semivolatile hydrocarbons were present in the test solutions as polar compounds, with chromatographic information confirming the signal was primarily from biodegradation metabolites (rather than natural organic matter). Also detected in some test solutions were BTEX compounds, at concentrations up to 14 µg/L (Total BTEX, Table 3), most of which was attributed to toluene (Supporting Information Table S2). Concentrations of individual hydrocarbon compounds including toluene, naphthalene, and benzene in test solutions were below current Australian and New Zealand water quality default guideline values for marine waters (ANZG 2018). Concentrations of N were above laboratory limit of reporting in most test solutions, with ammonia and nitrate concentrations elevated in some test solutions to maximum values of 27.4 and 20.2 mg/L, respectively (Table 3). These values are well in excess of the current default guideline value of 0.91 mg/L for total ammonia in marine waters (for 95% protection; ANZG 2018). Although the source of higher nutrient concentrations may in part be attributed to input from wildlife (Erskine et al. 1998), a likely contributing source is residual nutrients from additions during the remediation process. This latter source of nutrients is particularly the case for TS1, TS2, and TS6, which are comprised of samples taken from piezometers positioned in bedrock and therefore are more likely to retain the chemical signature of past remediation activities (i.e., nutrient addition) due to long residence times. This signature includes elevated ammonia, which is likely to have been preserved under the relatively anaerobic conditions of the bedrock substratum. Anaerobic conditions are common in aged hydrocarbon groundwater plumes, given that early‐phase aerobic bacterial degradation of hydrocarbons eventually converts to anaerobic conditions. Under reducing anaerobic conditions, the nitrification of ammonium to nitrate is suppressed (Böhlke et al. 2006).

**Table 3:** Selected physicochemical parameters measured in test solutions (TS1–TS7) and controls (SW and HSB) in 2 rounds of testing (R1 and R2) and toxicity observed in Microtox tests^a^

Test solution		Concentration (%)^b^	TRH, >C10–C40		Total BTEX (µg/L) LOR: 1	Nutrients (as N)		Microtox EC50 (%)^c^
			TRH (µg/L)	TRH‐SG (µg/L)		Ammonia (mg/L)	Nitrate (mg/L)	
			LOR: 100	LOR: 100		LOR: 0.01	LOR: 0.01	
SW	R2	100	<100	<100	<1	0.24	0.17	NMT
HSB	R1	78	<100	<100	<1	0.08	0.30	NMT
	R2	86	<100	<100	<1	0.10	0.18	NMT
TS1	R1	79	280	<100	<1	21.9	0.36	NMT
	R2	87	280	<100	<1	27.4	0.25	40
TS2	R1	78	650	<100	1	5.97	0.15	NMT
	R2	87	760	<100	<1	8.97	0.44	24
TS3	R1	76	290	<100	9	1.67	1.95	NMT
	R2	86	450	<100	<1	1.93	2.07	NMT
TS4	R1	79	350	<100	<1	2.05	5.09	89
	R2	88	280	<100	<1	1.74	16.1	92
TS5	R1	78	<100	<100	8	12.4	20.2	NMT
TS6	R1	79	<100	<100	<1	26.9	2.81	NMT
	R2	87	<100	<100	<1	21.4	5.50	NMT
TS7	R1	81	810	<100	14	1.69	0.26	NMT
	R2	86	670	<100	2	1.58	0.19	NMT

HSB = hypersaline brine; LOR = limit of reporting; NMT = no measurable toxicity; SG = using silica gel cleanup; SW = seawater; Total BTEX = sum of benzene, toluene, ethylbenzene, xylenes (meta‐, para‐ and ortho‐); TRH = total recoverable hydrocarbon; TS = test solution.

^a^ The full suite of measured physicochemical parameters is provided in Supporting Information Table S2.

^b^ % original sample, post HSB addition for salinity adjustment to ambient SW of 33‰–34‰.

^c^ Toxicity interpretation: EC50 ≤ 25% extremely toxic; 25%–50% highly toxic; 51%–75% moderately toxic; 76%–100% slightly toxic; >100% NMT.

### Response matrix assessment

**Figure 2:**
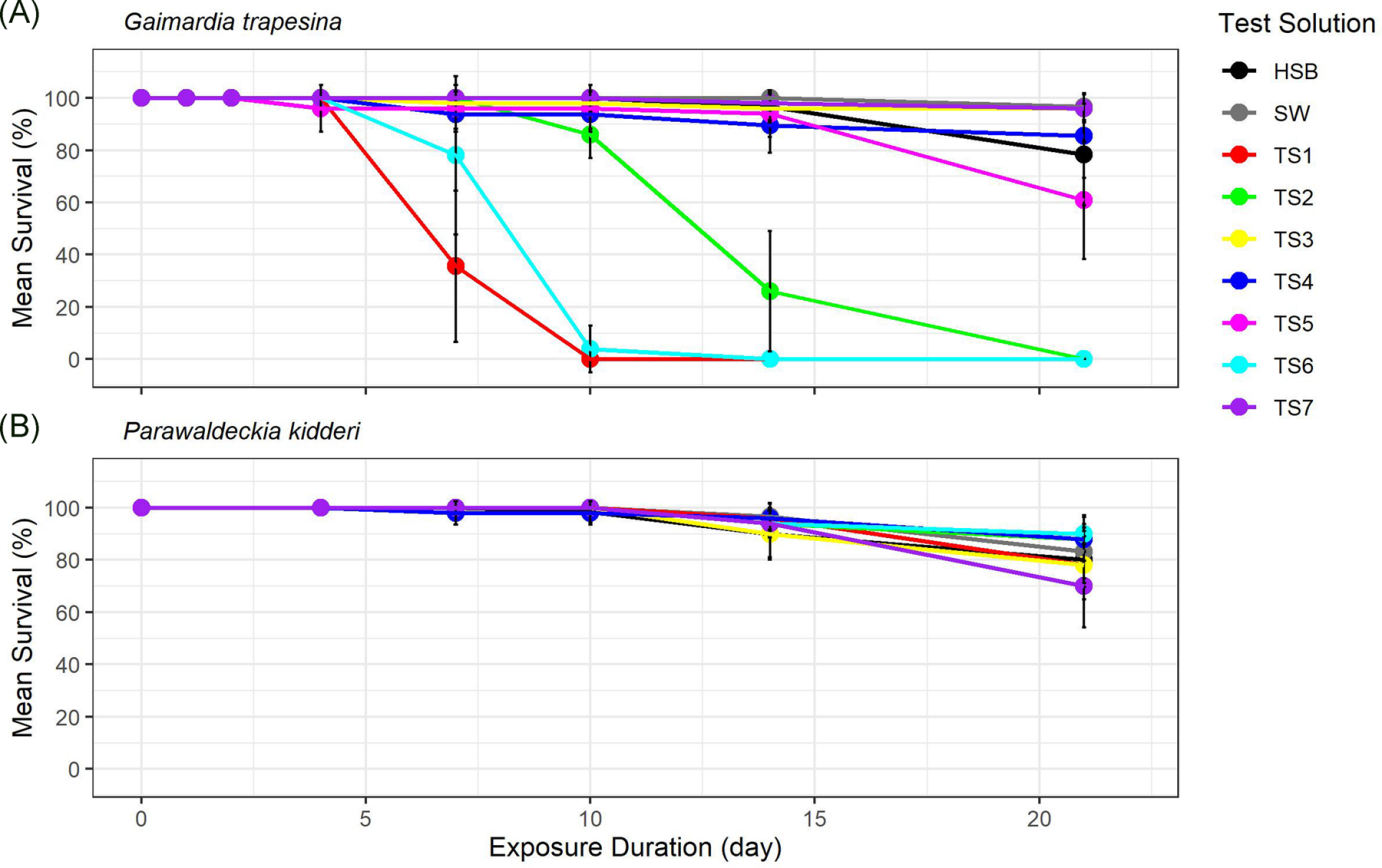
Survival of marine invertebrates exposed to test solutions (TSs). Two species with different responses are shown as examples: bivalve *Gaimardia trapesina*, Round 1 (a relatively sensitive species) (**A**); amphipod *Parawaldeckia kidderi*, Round 2 (a relatively tolerant species) (**B**). Data are mean ± SD (*n* = 6 for controls and 5 for test solutions); test duration was 21 d. A figure showing all 11 species and 2 test rounds is provided in Supporting Information Figure S1.

**Figure 3:**
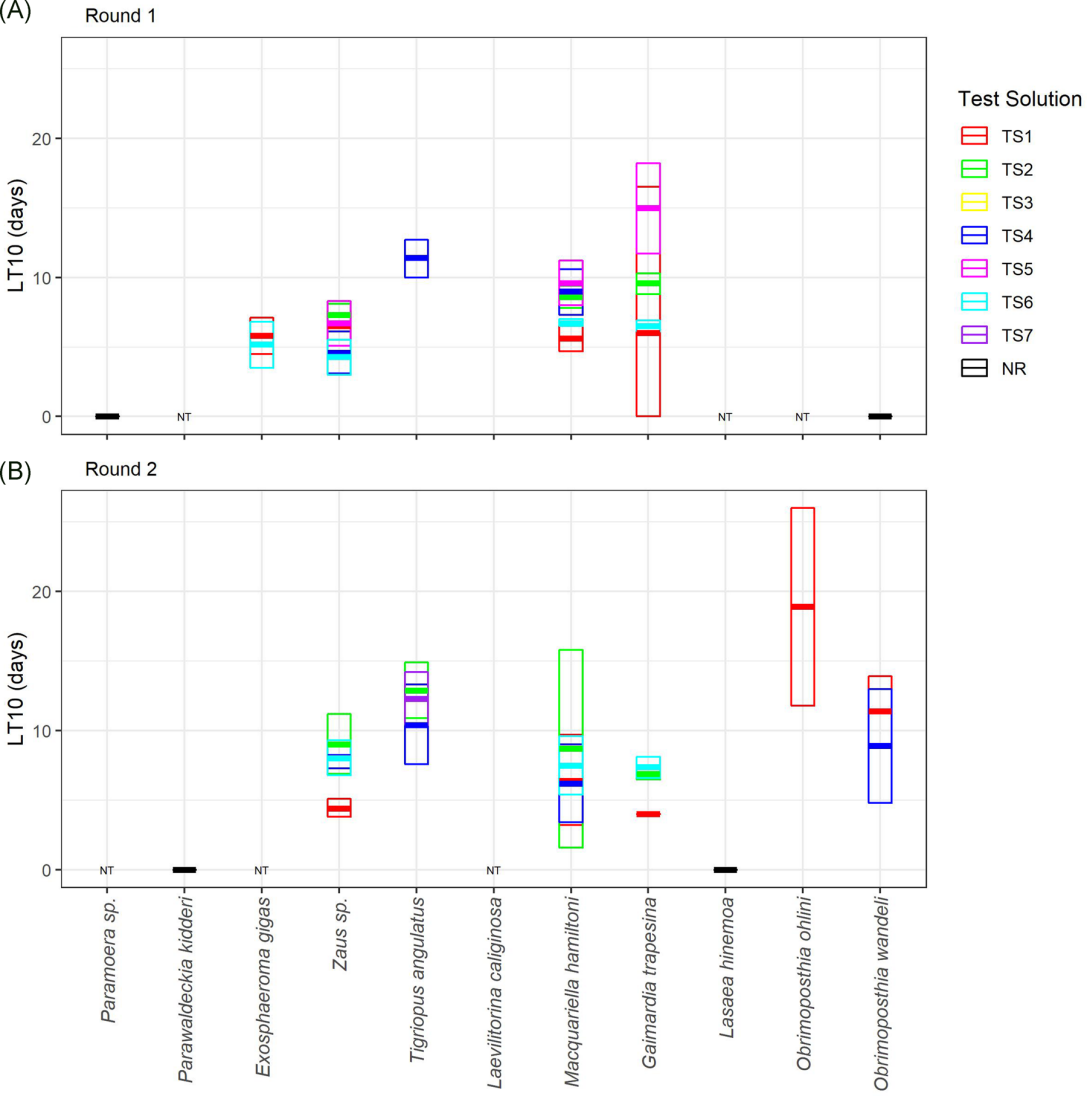
Summary of species response to test solutions (TSs) in test Rounds 1 (**A**) and 2 (**B**). Modeled LT10 estimates represent the lethal time taken to reach a 10% decrease in survival. Thicker horizontal lines indicate LT10, and thinner horizontal lines are upper and lower 95% confidence intervals. Only combinations (species–test solution) in which a response was determined are shown. NR = no response in all test solutions; NT = not tested.

The response matrix we present provides a methodical and transparent process to assess response for the 110 test combinations (8 species × 7 test solutions in Round 1, 9 species × 6 test solutions in Round 2). This procedure relies on interrogation of the raw data (Figure 2, Supporting Information Figure S1) and modeled LT data (Supporting Information Table S3 and Figure S2). We provide full data within the Supporting Information tables and figures, with examples or data subsets in which a response was deemed to occur shown within the manuscript. Modeled estimates of sensitivity (i.e., LT10 and LT50 and confidence limits) for test species–test solution combinations in which a response was deemed to occur (after applying the expert judgment response matrix) are shown in Figure 3 and Table 4.

**Table 4:** Modeled lethal time estimates (days) causing a 10% (LT10) and 50% (LT50) decrease in survival for species–test solution–test round combinations in which a response was determined using the response matrix

Species	Test solution	Test round	Test duration (d)	LT10	LL	UL	LT50	LL	UL
*Exosphaeroma gigas*	TS1	1	14	5.8	4.5	7.1	14.6	12.8	16.3
	TS6	1	14	5.2	3.5	6.8	13.9	11.8	16.0
*Zaus* sp.	TS1	1	14	6.5	6.1	6.8	8.0	7.6	8.3
	TS1	2	14	4.4	3.8	5.1	5.6	5.1	6.0
	TS2	1	14	7.3	6.6	8.1	10.7	10.1	11.2
	TS2	2	14	9.0	6.9	11.2	10.3	9.6	11.0
	TS4	1	14	4.6	3.1	6.1	9.5	8.3	10.7
	TS4	2	14	8.1	7.3	9.0	11.7	11.2	12.3
	TS5	1	14	6.7	5.1	8.3	10.1	9.2	10.9
	TS6	1	14	4.3	3.0	5.5	7.7	6.9	8.5
	TS6	2	14	8.0	6.8	9.3	10.2	9.7	10.7
*Tigriopus angulatus*	TS2	2	21	12.9	10.9	14.9	26.0	22.6	29.5
	TS4	1	21	11.4	10.0	12.7	15.7	14.9	16.4
	TS4	2	21	10.4	7.6	13.3	20.8	17.8	23.9
	TS7	2	21	12.3	10.4	14.2	29.2	24.0	34.4
*Laevilitorina caliginosa*	TS1	2	21	12.0	10.4	13.7	18.9	17.7	20.1
	TS2	2	21	15.0	12.7	17.3	21.4	20.4	22.5
*Macquariella hamiltoni*	TS1	1	14	5.6	4.7	6.5	7.1	6.9	7.4
	TS1	2	14	6.4	3.2	9.7	7.1	6.5	7.7
	TS2	1	14	8.6	7.8	9.4	12.0	11.5	12.5
	TS2	2	14	8.7	1.6	15.8	9.8	8.4	11.1
	TS4	1	14	9.0	7.3	10.6	14.6	13.3	15.8
	TS4	2	14	6.2	3.4	9.0	12.6	9.9	15.2
	TS5	1	14	9.6	8.0	11.2	14.7	13.5	15.8
	TS6	1	14	6.7	6.5	7.0	8.4	8.2	8.6
	TS6	2	14	7.5	5.4	9.6	10.4	9.4	11.3
*Gaimardia trapesina*	TS1	1	21	6.0	−4.5	16.5	6.8	4.3	9.3
	TS1	2	21	4.0	4.0	4.0	4.2	3.1	5.3
	TS2	1	21	9.6	8.8	10.3	12.4	11.9	12.9
	TS2	2	21	6.9	6.5	7.3	8.3	7.9	8.6
	TS5	1	21	15.0	11.7	18.2	22.9	20.5	25.3
	TS6	1	21	6.5	6.1	6.9	7.7	7.3	8.2
	TS6	2	21	7.4	6.6	8.1	8.9	8.5	9.4
*Obrimoposthia ohlini*	TS1	2	21	18.9	11.8	26.0	22.7	16.3	29.2
*Obrimoposthia wandeli*	TS1	2	21	11.4	8.8	13.9	19.0	17.0	21.1
	TS4	2	21	8.9	4.8	13.0	48.7	7.6	89.8

LL = lower 95% confidence limit; LT = lethal time; TS = test solution; UL = upper 95% confidence limit. Modeled estimates for all combinations are provided in Supporting Information Table S3.

The full response matrix is shown in Supporting Information Table S4 and an excerpt of this, as an example of the process, is shown in Table 5 for 2 species. For the relatively sensitive species, *G. trapesina*, 4 of the 7 test solutions were deemed as having a response (response score >65%). For 3 of these test solutions, the 5 response criteria scored 1 (response). Interpretation of TS5 was more complex, however, and provides an example of this assessment process. Criterion 2 had a borderline rating due to the difference in response between HSB control and test solution being 15% to 20% (Table 5), and Criteria 4 and 5 had a no response rating due to LT10 and LT50 estimates overlapping with the HSB control estimate range (Supporting Information Figure S2 and Table S3). The relatively tolerant *Parawaldeckia kidderi* was found to show no response to any test solution, with a maximum response score of 41% calculated for TS7.

**Table 5:** An excerpt of the response matrix showing 2 species with differing responses as examples^a^

Species	Test solution	Mean survival at test end (%)							Response score (%)^c^	Overall expert judgment^d^
				
		Test solution	Difference^b^	Response criteria						
				1	2	3	4	5		
*Gaimardia trapesina*	TS1	0	78	1	1	1	0	1	93	Response
	TS2	0	78	1	1	1	1	1	100	Response
	TS3	96	–18	0	0	0	0	0	0	—
	TS4	85	–7	0	0	0.5	0	0	9	—
	TS5	61	18	1	0.5	1	0	0	68	Response
	TS6	0	78	1	1	1	1	1	100	Response
	TS7	96	–18	0	0	0	0	0	0	—
*Parawaldeckia kidderi*	TS1	78	2	0	0	1	0	0	18	—
	TS2	88	–8	0	0	0	0	0	0	—
	TS3	78	2	0	0	1	0	0	18	—
	TS4	88	–8	0	0	0	0	0	0	—
	TS6	90	–10	0	0	0	0	0	0	—
	TS7	70	10	0.5	0	1	0	0.5	41	—

HSB = hypersaline brine; TS = test solution.

^a^ Species shown are those also shown in Figure 2: *G. trapesina*, Round 1, relatively sensitive species; *P. kidderi*, Round 2, relatively tolerant species. The full response matrix for all species is provided in Supporting Information Table S4. The 5 response criteria were scored for each species and test solution (TS1–TS7) using the criteria described in Table 2. Mean survival at test end (for test solution and HSB control minus test solution) are used for Criteria 2 and 3. Each criterion was scored 1 if met (response), 0 if not met (no response), or 0.5 if borderline or unclear.

^b^ Difference in mean survival at test end between HSB control and test solution, where HSB control for *G. trapesina* = 78% and *P. kidderi* = 80%.

^c^ The calculated response score (%) is the sum of the 5 criteria scores, each multiplied by their weighting value (defined in Table 2) and expressed as a percentage.

^d^ Test solutions with response scores of 65% or greater were deemed as toxic (overall expert judgment = Response).

From the full response matrix showing all 110 test solution–species combinations, toxicity was observed in approximately one‐third of combinations (36 cases with response scores >65%). Fourteen of these 36 cases had response scores of 100%, and these were almost exclusively in 2 of the most sensitive species, *Zaus* sp. and *G. trapesina* (Supporting Information Table S4). Of the remaining 74 cases that showed no response, 39% had a response score of 0% (Supporting Information Table S4). A summary of responses for all test species–test solution combinations is provided in Table 6.

**Table 6:** Response summary of the relative toxicity of test solutions (TS1–TS7) and sensitivity of test species, determined in 2 test rounds (R1 and R2), derived from the expert judgment response matrix (Supporting Information Table S4)^a^

Species	TS1		TS2		TS3		TS4		TS5		TS6		TS7		% toxic response
	R1	R2	R1	R2	R1	R2	R1	R2	R1	R2	R1	R2	R1	R2	
*Paramoera* sp.	—		—		—		—		—		—		—		0
*Parawaldeckia kidderi*		—		—		—		—				—		—	0
*Exosphaeroma gigas*	R		—		—		—		—		R		—		29
*Zaus* sp.	R	R	R	R	—	—	R	R	R		R	R	—	—	69
*Tigriopus angulatus*	—	—	—	R	—	—	R	R	—		—	—	—	R	31
*Laevilitorina caliginosa*	—	R	—	R	—	—	—	—	—		—	—	—	—	15
*Macquariella hamiltoni*	R	R	R	R	—	—	R	R	R		R	R	—	—	69
*Gaimardia trapesina*	R	R	R	R	—	—	—	—	R		R	R	—	—	54
*Lasaea hinemoa*		—		—		—		—				—		—	0
*Obrimoposthia ohlini*		R		—		—		—				—		—	17
*Obrimoposthia wandeli*	—	R	—	—	—	—	—	R	—		—	—	—	—	15
Microtox test	—	R	—	R	—	—	R	R	—		—	—	—	—	31
% toxic	58		47		0		47		33		37		5		

R = response; TS = test solution; — = no response; empty cell = not tested.

^a^ Overall response summary values of % toxic (for test solutions) and % toxic response (for species) are shown and represent the proportion (%) response out of total number tested.

### Relative toxicity of test solutions and relative sensitivity of test species

Response of biota to test solutions was time dependent and variable between the 11 test species, the 7 test solutions, and the 2 rounds (Supporting Information Figure S1). Some species were sensitive to some test solutions, with survival decreasing through time (e.g., *G. trapesina*, Figure 2). In other more tolerant species, no response was observed in any test solutions for the entire test duration (e.g., *Parawaldeckia kidderi*, Figure 2). For all test solutions in which a response was assessed, mortality was not observed until ≥4 d of continuous exposure (Supporting Information Figure S1). This delayed response was also supported through modeled estimates, representing the time at which 10% mortality occurred (range 4.0–18.9 d) and 50% mortality occurred (range 4.2–48.7 d; Table 4).

Three of the 11 species (*Paramoera* sp., *Parawaldeckia kidderi*, *Lasaea hinemoa*) were insensitive to exposure, showing no response in any test solution (Supporting Information Figure S1, Table 6). In contrast, *Zaus* sp., *M. hamiltoni*, and *G. trapesina* were the most sensitive species, with responses observed in more than 50% of cases (Table 6, Figure 3). Toxicity detected in Microtox assays generally correlated with responses observed in invertebrate tests. Where there was a response in Microtox, there was also a minimum of 3 and up to 6 invertebrate species also responding (Table 6). However, there were exceptions; for example, TS5 and TS6 were not toxic to Microtox but were toxic to several invertebrates. Microtox appears to be a moderately sensitive indicator of toxicity (responses observed in 31% of cases, Table 6). However, 2 of the 4 responses noted for Microtox (TS4 in Round 1 and Round 2, Table 6) were classified as only slightly toxic (Table 3). These differences in sensitivity between invertebrate test species, and the lack of sensitivity of the Microtox assay in some cases, highlight the need to include multiple local species from a range of taxa to properly assess the impacts of contaminants on a site‐specific basis.

The most toxic test solution was TS1, with responses in 58% of cases (Table 6). The least toxic was TS3, which elicited no response in any species tested, including Microtox (Table 6). The relative order of toxicity was TS1 > TS2 = TS4> TS6 > TS5 » TS7 > TS3. There was some difference in the toxicity of test solutions between rounds, with most showing greater toxicity in Round 2 (Table 6). This is likely due to the fact that some species were tested over only 14 d in Round 1 and showed no response, but when test duration was extended to 21 d in Round 2, a response was observed. There were also some differences in the concentration of test solutions due to the addition of different volumes of HSB solutions required to achieve the test salinity of 33‰ to 34‰, with final test solutions after salinity adjustment up to 10% more concentrated in Round 2 than in Round 1 (Table 3). Differences in toxicity of test solutions between rounds is also expected due to temporal variation in groundwater dynamics at the site, which are influenced by rainfall, tides, et cetera.

### Relationship between observed toxicity and groundwater physicochemistry

There were no definitive trends in toxicity associated with the physicochemical parameters or chemical compositions of the test solutions measured in the present study, and toxicity could not be attributed to compounds of petrogenic origin. The TRHs were identified as being predominantly polar compounds and primarily biodegradation metabolites (Table 3), which have been recognized as potential contributors to toxicity in aged fuels (Idowu et al. 2019). Our results, however, do not support this, and instead agree with studies that suggest that once a spill is highly degraded, toxicity may be relatively low (Zemo et al. 2017; Patterson et al. 2020). In both the present study and that of Patterson et al. (2020), metabolites in groundwater from degraded fuel‐contaminated sites did not illicit a clear toxic effect to a range of aquatic biota. Concentrations of polar compounds in the present study were relatively low (maximum of 810 µg/L, Table 3). In contrast to a fresh spill, we would expect equivalent dissolved concentrations of undegraded nonpolar petrogenic hydrocarbons to elicit a different, and potentially stronger, toxicity response. Toxicity data based on terrestrial species from Macquarie Island support this expectation, with soil containing fresh fuel product eliciting higher toxicity than soil with aged fuel contamination (Mooney et al. 2013).

Test solution 7 had the highest concentration of polar and BTEX compounds (Table 3), yet was relatively nontoxic, with a response observed in only 1 of 19 tests (5% of cases, Table 6). In contrast, TS2 had a similar polar compound concentration (Table 3) but was the second most toxic (47% of cases). TS1 was the most toxic (Table 6) and had low hydrocarbon concentrations but elevated ammonia (Table 3). The only other test solution that had elevated ammonia and low polar hydrocarbon concentration (Table 3) was TS6, which displayed midrange toxicity (37% of cases, Table 6). Given that the ammonia concentrations were generally in excess of default guideline values (ANZG 2018), some observed toxicity is likely attributed to ammonia.

Samples for composite test solutions were selected on the basis of their previous contaminant history rather than on spatial location, in an attempt to obtain a range and gradient of chemical test solutions. Although the data obtained were therefore not suitable for elucidating spatial patterns of toxicity across the site, nor for identifying potential hotspots needing additional management, the 2 methods used to sample groundwater may provide some information on the vertical gradient through the substrata (soil to bedrock). Test solutions created using deeper groundwater obtained from piezometers installed in bedrock, as was the case for Fuel Farm piezometers, were consistently more toxic than those created from groundwater taken from seeps at the higher elevation soil–bedrock interface. Groundwater from piezometers with long residence times provides insights into past conditions, whereas groundwater seeps with shorter residence times were more representative of current conditions. The greater toxicity of test solutions prepared using samples from piezometers versus those from seeps is not adequately explained by hydrocarbon concentrations or other water quality indicators measured in the present study, with the potential exception of ammonia (Table 3). For example, TS1, TS2, and TS6 (groundwater taken from Fuel Farm bedrock piezometers) showed higher concentrations of ammonia and were more toxic relative to test solutions prepared from seep‐sourced groundwater across the same area (TS3 and TS7; Table 6). It was also noted that groundwater from piezometers at the Fuel Farm, prior to composite test solution preparation, had lower DO and redox potential compared with the more oxidizing conditions measured at the seeps (data not shown). Historical hydrocarbon‐impacted groundwater plumes commonly exhibit reducing (anoxic) conditions, which suppress the nitrification of ammonium to nitrate (Böhlke et al. 2006). Based on existing data, it is not clear whether this process is causing the difference in observed ammonia concentration between seeps and piezometers at the Fuel Farm. Other unmeasured chemical or physical characteristics associated with hydrogeological differences may also exist between these groundwater types and may drive the observed toxicity.

Although the drivers of toxicity in groundwater were inconclusive, identified differences in the composition of samples and toxicity to test species could be further interrogated and tested (e.g., specific nutrient or hydrocarbon components), and then used to inform which contaminants are potentially most toxic and therefore of greatest concern. This information would help direct remediation efforts by identifying contaminants that we should focus on reducing or removing in the remediation process.

### Rationale for experimental design

In the present study, we used time–response models to test the response of biota to a single maximum concentration sample over time as an alternative to traditional concentration–response models. Lethal time estimates generated were then used to directly compare test solution and species to determine relative toxicities. This methodology was adopted both for practical reasons and to allow for better interpretation of the risk posed by groundwater discharges to the natural environment at Macquarie Island. Such an approach allowed us to maximize the number of groundwater samples that could be assessed (as composite test solutions) and the number of local species that could be tested within a finite field season in a remote location. Because maximum concentration test solutions in many cases showed no effect on biota, lethal concentration estimates would not have been able to be generated if a dilution series had been tested. Furthermore, because the aim of the present study was to determine the risk of groundwater at this specific site, it was not our intention to generate sensitivity values that could be incorporated into a species sensitivity distribution model to generate protective or hazardous concentrations for broader application. Hence traditional concentration–response testing would not have generated any further useful data to inform the present study. In addition, in the absence of dilution dynamics data for the coastline at Macquarie Island, lethal concentration data could not have been directly applied to spatial risk modeling. Because the overall aim of the present study was to determine the relative risk of groundwater discharges in the context of the receiving environment, determining the time to affect communities under a worst‐case scenario, with minimal dilution of test solutions, provided the most useful information.

### Critique of response matrix and expert judgment approach

We used a mix of raw and modeled data, coupled with expert judgment, to provide a quantitative assessment of biological response. By using multiple lines of evidence to fully interrogate data, we can more accurately predict the response of biota, particularly in the case of partial responses, as opposed to relying on modeled response estimates in isolation. Multiple lines of evidence criteria were weighted using expert judgment. Visual inspection of response plots to interpret raw data (Criterion 1) was weighted highly because this characterizes the actual biological response to test solution as compared to controls. Low weighting was ascribed to modeled estimates (Criteria 4 and 5) because they did not always follow the classic dose–response curve and were able to be produced for all data sets, including partial or marginal responses, leading to a high level of uncertainty.

From the 110 test solution–species combinations tested, 69 showed a clear outcome (14 response and 55 no response) based on modeled LT10 and LT50 estimates alone (Criteria 4 and 5, Supporting Information Table S4). In these situations, no additional decision framework was required. The remaining 41 cases, however, were less clear, and our ability to interpret response was greatly enhanced through the use of the response matrix. In particular, there were 21 cases in which a response was evident from only 1 of the modeled estimates (either LT10 or LT50). In these partial response cases, Criteria 1 to 3 (Supporting Information Table S4) were particularly important to further interrogate data, enabling more thorough, evidence‐based decision making to assess response. Some other cases showed no evidence of response from modeled estimates (largely due to suboptimal controls), but were assessed as toxic based entirely on Criteria 1 to 3. The rigor of this holistic response matrix provides a precautionary approach to capture all possible responses, allowing a conservative stance to be adopted where appropriate.

This approach is thorough and transparent, and creates greater certainty of accurately deeming a sample to be toxic or nontoxic by using multiple criteria, rather than relying solely on the outcomes of statistical models (i.e., LC*x* comparisons) as is typical of ecotoxicological assessments. This approach was in part necessary because partial or minimal responses were often observed, resulting in a poor fit of data to dose–response models. Although modeled estimates could still be calculated in these cases, they had low confidence (i.e., large CIs) and were often well outside testing durations (Supporting Information Figure S2). Reliance on such poor sensitivity estimates would result in high uncertainty in the protection of aquatic ecosystems and risk management decisions. Sensitivity estimates alone were therefore not useful in deciding whether an actual response was observed in the present study, thereby hindering predictions of likely impacts in the field. The expert judgment response matrix approach we present provides a detailed and systematic framework that can be used in future studies in which traditional dose–response models provide a poor fit to response data.

### Risk assessment of impacts of groundwater discharges

Results from toxicity assessments and on‐site observations provide a strong basis of evidence to assess the impacts of groundwater discharges from terrestrial contaminated sites into the environment at Macquarie Island. The majority of test solutions showed low toxicity to the suite of species tested. Although some test solutions were toxic to some test biota after a minimum of 4 d exposure, these were under the worst‐case scenario conditions, with tests on “undiluted” samples with continuous exposure over extended periods. These conditions are highly unlikely to ever occur naturally. In the field, the likelihood of extended exposures that would result in mortality is low, with marine biota potentially exposed to only low concentrations of contaminants in diluted groundwater for only short periods of time.

Under real field conditions, groundwater discharges enter the coastal marine environment at a highly variable rate, which is dependent on microscale changes to the hydraulic conductivity of the bedrock formation coupled with constantly changing tidal and precipitation influences. Strong Southern Ocean wave action and tidal influences in the high‐energy receiving environment ensure immediate dilution and rapid dispersal of contaminants in groundwater discharges, and prevent the occurrence of hotspots of contamination in intertidal through to subtidal zones. In addition, few receptor marine organisms actually occur in the upper coastal splash zones where groundwater discharges before being dispersed across the intertidal zone. This intertidal zone is comprised of rock platforms and rocky substrates of pebble and coarse sand, with little to no fine sediment to act as a sink for contaminants. In such a receiving environment, the capacity for sorption and concentration of contaminants and resuspension and reexposure to biota via the substrate is also very low.

Given the generally low toxicity of test solutions over prolonged exposures, the low discharge rates of contaminated groundwater, the high‐energy receiving environment, and the high rainfall and therefore rapid dilution and dispersal, the overall likelihood of risk of impacts from contaminated groundwater to marine communities at Macquarie Island is deemed low.

## CONCLUSION

The present study has provided critical information that can be employed to inform management decisions at Macquarie Island. Our response criteria ensured a conservative approach to assessing risk, as is appropriate for this high‐value World Heritage‐listed site. Although toxicity was observed in response to test solutions in a number of species tested, the likelihood of these groundwater discharges causing an effect on communities in the receiving environment is negated by the unrealistic long press‐exposure scenarios under which responses were observed in the present study. The minimum duration of constant exposure required to elicit a response was 4 d. Such a prolonged and continuous exposure is highly unlikely to ever occur given the low discharge rates of contaminated groundwater entering the high‐energy coastal environment at Macquarie Island. Although some petroleum contamination remains in soils at the Fuel Farm and Power House sites at Macquarie Island, the present study provides evidence that contaminated groundwater discharging from these sites is unlikely to represent a toxicity risk to the coastal marine communities tested. We therefore suggest, based on conditions at the time of testing, that no further site management is required to reduce risks from existing remediated contaminated sites to the marine environment.

## DISCLAIMER

There are no conflicts of interest associated with this work.

### Open Data and Materials Badges

This article has earned Open Data and Open Materials Badges for making publicly available the digitally shareable data and components of the research methodology necessary to reproduce the reported results, procedure and analysis. The data and materials are available at https://doi.org/10.4225/15/516F550D98175. Learn more about the Open Practices badges from the Center for Open Science: https://osf.io/tvyxz/wiki.

## SUPPLEMENTAL DATA

**Figure S1.** Survival of 11 marine invertebrate species exposed to test solutions (TS1–TS7) from remediated fuel spill sites at Macquarie Island in the 2 rounds of toxicity tests.

**Figure S2.** LT10 and LT50 modeled estimates (±95% CI) for each marine invertebrate species in the 2 rounds of toxicity tests with 7 test solutions (TS1–TS7) from remediated fuel spill sites at Macquarie Island.

**Table S1.** Summary of groundwater discharge collection for preparation of composite test solutions (TS1–TS7) for testing in Rounds 1 and 2, and previous chemical characterization

**Table S2.** Physicochemical parameters measured in test solutions (TS1–TS7) and controls (SW and HSB) for the 2 test rounds

**Table S3.** Modeled lethal time estimates of time (d) causing a 10% (LT10) and 50% (LT50) decrease in survival

**Table S4.** Response matrix used to assess the toxicity of 7 test solutions (TS1–TS7) to 11 test species in 2 test rounds.

## Supporting information

This article contains online‐only Supporting Information.

Supporting information.

Supporting information.

## Data Availability

All data associated with this study are publicly available from the Australian Antarctic Data Centre, https://doi.org/10.4225/15/516F550D98175, which includes reproducible code used for analysis and visualization of data.
