# Supplementary material for: Using an expert judgment response matrix to assess the risk of groundwater discharges from remediated fuel spill sites to the marine environment at sub‐Antarctic Macquarie Island, Australia
Source: Integr Environ Assess Manag. 2021 Feb 10;17(4):785–801. doi: 10.1002/ieam.4382 (PMC8359375; doi:10.1002/ieam.4382)

Supplementary Figures

Figure S1. Survival of 11 marine invertebrate species exposed to test solutions (TS1-7) from remediated fuel spill sites at Macquarie Island in the 2 rounds of toxicity tests. Plots show percent survival over 14 or 21 day exposure. Data are mean ± SD (n = 6 for controls and 5 for test solutions).

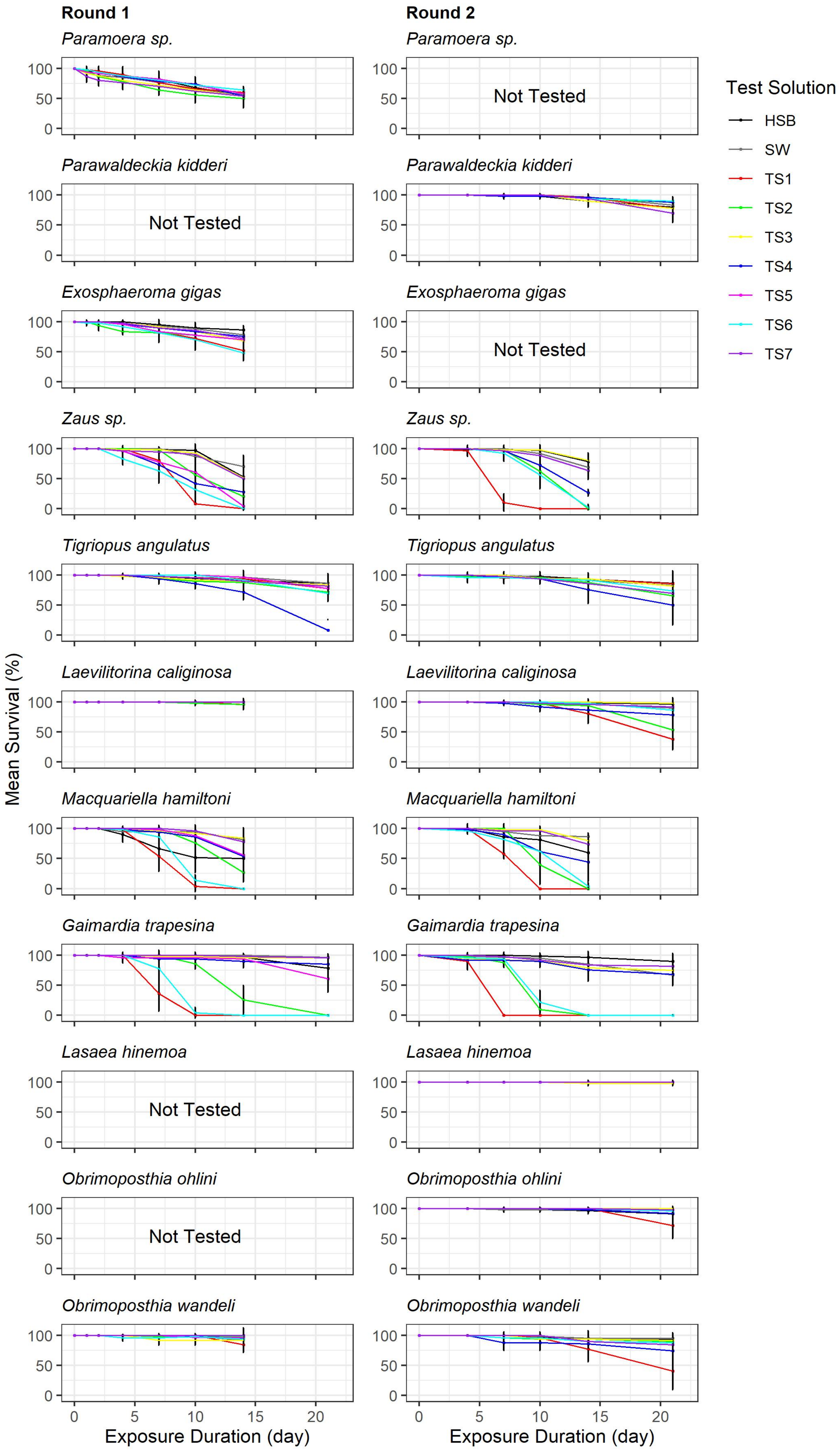

Supplementary Figures

Figure S2. LT10 and LT50 modelled estimates (±95% CI) for each marine invertebrate species in the two rounds of toxicity tests with seven test solutions (TS1-7) from remediated fuel spill sites at Macquarie Island. Coloured bars represent TS1-7; with bars extending to lower and upper 95% confidence limits. Horizontal reference lines show HSB control; solid line = LT estimate, dashed lines = 95% CI. Note figure includes all estimates obtained via dose response modelling, of which some are deemed no response using the expert judgement response matrix (Supplementary Table S4). No data shown for: tests in which survival remained at 100% throughout test duration (no modelled estimate obtained; *Laevilitorina caliginosa* R1 all test solutions except TS2, *Lasaea hinemoa* R2 all test solutions except TS3 and *Obrimoposthia wandeli* R1 TS7) and for R2 TS5 (not tested). Cases in which confidence intervals were excessively wide are marked with * (see Supplementary Table S3 for data).

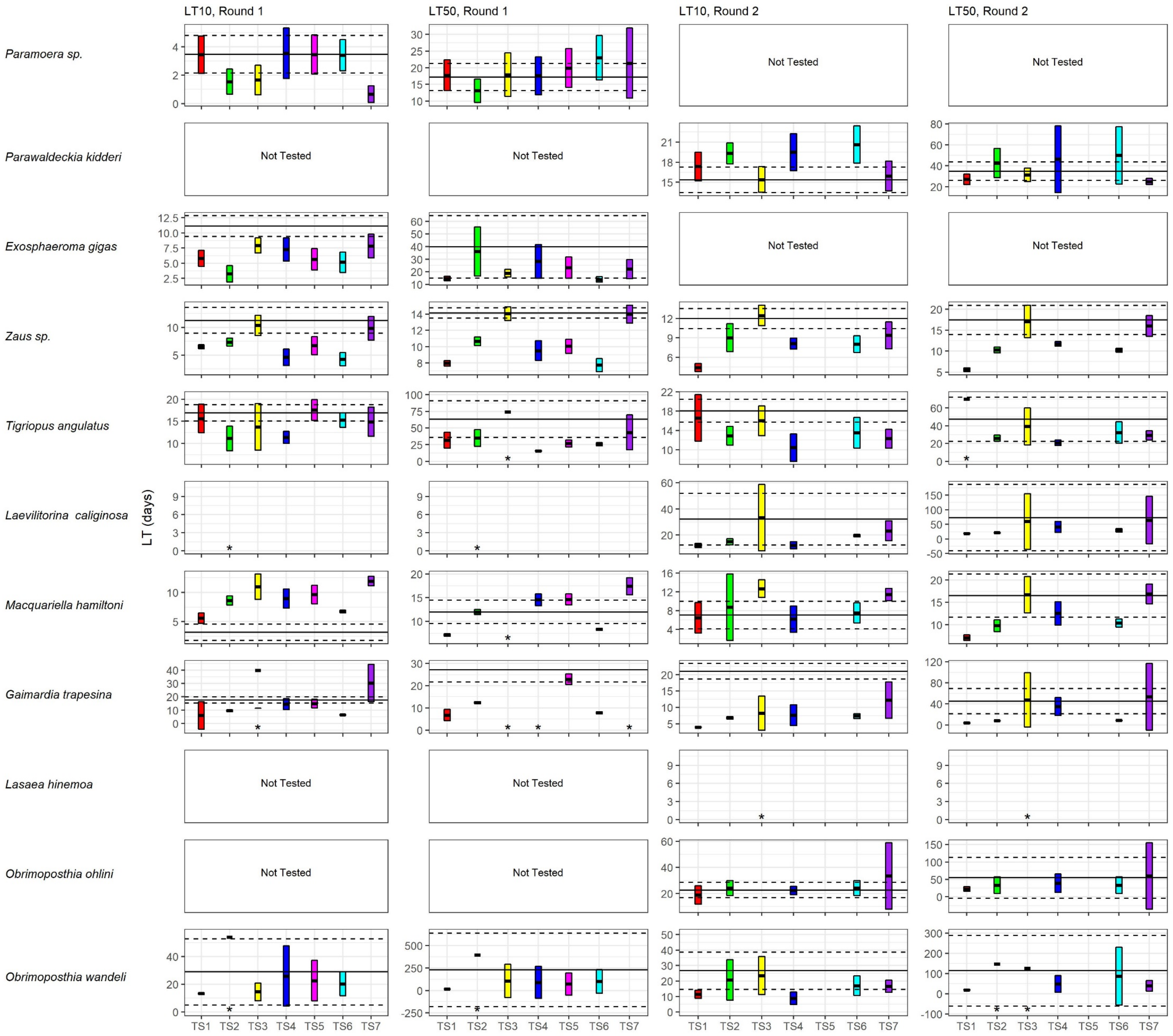

Supplement: Supplementary file 1 — Supporting information. [file IEAM-17-785-s001.pdf]
